# Targeting EGFR Induced Oxidative Stress by PARP1 Inhibition in Glioblastoma Therapy

**DOI:** 10.1371/journal.pone.0010767

**Published:** 2010-05-24

**Authors:** Masayuki Nitta, David Kozono, Richard Kennedy, Jayne Stommel, Kimberly Ng, Pascal O. Zinn, Deepa Kushwaha, Santosh Kesari, Frank Furnari, Katherine A. Hoadley, Lynda Chin, Ronald A. DePinho, Webster K. Cavenee, Alan D'Andrea, Clark C. Chen

**Affiliations:** 1 Department of Radiation Oncology, Dana-Farber Cancer Institute, Boston, Massachusetts, United States of America; 2 Harvard Radiation Oncology Program, Boston, Massachusetts, United States of America; 3 Almac Diagnostics, Craigavon, Northern Ireland, United Kingdom; 4 Department of Medical Oncology, Belfer Institute for Applied Cancer Science, Dana-Farber Cancer Institute, Boston, Massachusetts, United States of America; 5 Department of Neurology, Moores UCSD Cancer Center, University of California San Diego, La Jolla, California, United States of America; 6 San Diego Branch, Ludwig Institute for Cancer Research, La Jolla, California, United States of America; 7 Department of Genetics, University of North Carolina at Chapel Hill, Chapel Hill, North Carolina, United States of America; 8 Division of Neurosurgery, Beth Israel Deaconess Medical Center, Boston, Massachusetts, United States of America; University of Minnesota, United States of America

## Abstract

Despite the critical role of Epidermal Growth Factor Receptor (EGFR) in glioblastoma pathogenesis [1], [2], EGFR targeted therapies have achieved limited clinical efficacy [3]. Here we propose an alternate therapeutic strategy based on the conceptual framework of non-oncogene addiction [4], [5]. A directed RNAi screen revealed that glioblastoma cells over-expressing EGFRvIII [6], an oncogenic variant of EGFR, become hyper-dependent on a variety of DNA repair genes. Among these, there was an enrichment of Base Excision Repair (BER) genes required for the repair of Reactive Oxygen Species (ROS)-induced DNA damage, including poly-ADP ribose polymerase 1 (PARP1). Subsequent studies revealed that EGFRvIII over-expression in glioblastoma cells caused increased levels of ROS, DNA strand break accumulation, and genome instability. In a panel of primary glioblastoma lines, sensitivity to PARP1 inhibition correlated with the levels of EGFR activation and oxidative stress. Gene expression analysis indicated that reduced expression of BER genes in glioblastomas with high EGFR expression correlated with improved patient survival. These observations suggest that oxidative stress secondary to EGFR hyper-activation necessitates increased cellular reliance on PARP1 mediated BER, and offer critical insights into clinical trial design.

## Introduction

Historically, cancer therapeutic development has largely been driven by the principle of “oncogene addiction” – that cancer cells require increased activity of selected oncogenes and therefore tumor ablation can be achieved by inhibition of these oncogenes [7]. While “oncogene addiction”-based therapeutics have achieved notable successes in some cancers [7], their application to glioblastoma has yielded little efficacy. For instance, while EGFR mutations or copy number alterations are found in nearly 50% of all glioblastomas [1], [2], EGFR inhibition has yet to yield significant improvements in clinical outcome [3]. The ineffectiveness of such targeted therapy is explained in part by mutations in downstream signaling molecules [3] and redundant signaling from multiple co-activated receptor tyrosine kinases [8]. In this context, it is evident that meaningful therapy will require co-extinction of multiple oncogenes.

Emerging literature suggests an alternative strategy to the multi-target approach [4], [5]. These studies reveal that oncogene activation introduces secondary physiologic changes that stress cellular capacity for survival. Consequently, tumor cells become hyper-dependent on processes required to compensate for these stressful conditions. This phenomenon is termed “non-oncogene addiction” since the compensatory processes required for tumor survival are not oncogenic. As an example, RAS hyper-activation in colon cancer cells results in increased mitotic aberrancy and hyper-dependence on mitotic checkpoint function [5].

In this study, we explore the framework of “non-oncogene addiction” as it relates to oncogenic EGFR activation. As hyper-activation of several EGFR downstream effectors, including RAS and STAT3, elicits increased DNA damage accumulation [9], [10], [11], we tested whether the expression of a clinically pertinent EGFR oncogene, EGFRvIII [6], caused increased requirement for DNA repair as a form of non-oncogene addiction.

## Results and Discussion

### EGFRvIII over-expressing U87MG cells exhibited increased reliance on BER genes

**Figure 1:**
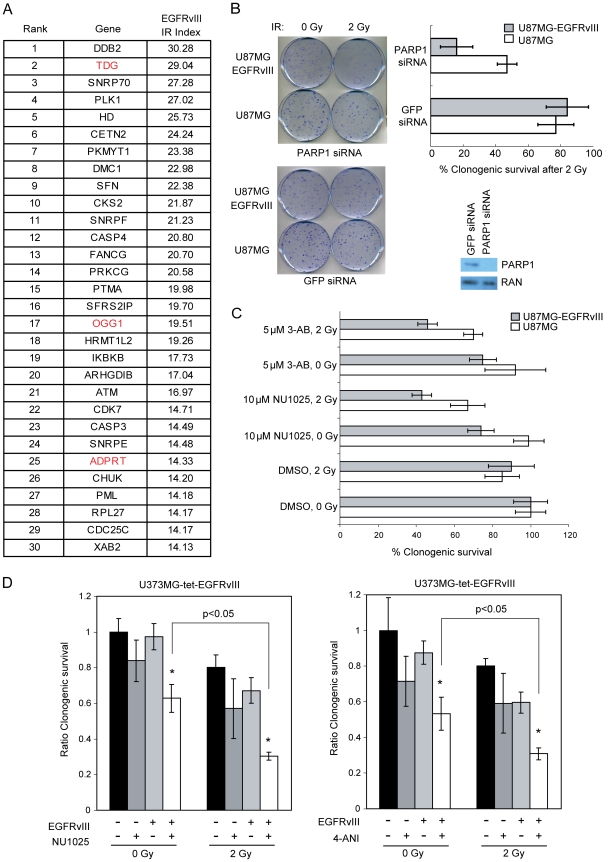
PARP1 inhibition preferentially radiosensitizes EGFRvIII hyperactive glioblastoma cells. (A) The top 30 siRNA targets that preferentially sensitized U87MG-EGFRvIII cells relative to parental U87MG cells. (B) PARP1 silencing preferentially radiosensitized EGFRvIII expressing U87MG, as measured by clonogenic survival (left and top right). PARP1 silencing efficiency (bottom right). (C) PARP1 inhibitors 3-AB and NU1025 radiosensitized EGFRvIII expressing U87MG. (D) PARP1 inhibitors NU1025 and 4-ANI preferentially radiosensitized U373MG cells expressing EGFRvIII. The U373MG cells harbor a tet-repressible EGFRvIII construct. EGFRvIII + denotes U373MG tet-EGFRvIII grown in the absence of doxycycline. EGFRvIII − denotes the cells grown in the presence of doxycycline. EGFRvIII expression levels were verified by Western blot (Fig. S3). Clonogenic survival after PARP1 inhibitor treatment was expressed as a ratio to DMSO treated cells. p-values were calculated using Student's t-test.

Given the mutually compensatory nature of many DNA repair pathways [12], [13], we reasoned that hyper-dependency on any particular DNA repair process would be most evident when cellular capacity for repair is saturated by exogenously introduced DNA damage. We selected Ionizing Radiation (IR) as a means of introducing DNA damage since IR is universally utilized in glioblastoma treatment. We adopted a siRNA screen-based approach, reasoning that silencing of genes required for the compensatory process might lead to preferential sensitization of a glioblastoma line over-expressing EGFRvIII (U87MG-EGFRvIII) relative to the parental line without such overexpression (U87MG). We screened a targeted siRNA library including 480 siRNAs directed against 240 DNA repair/damage response genes (Qiagen DNA repair subset v2.0). The top 30 candidates from this screen are shown in Fig. 1A.

Among the top candidate genes, we noted an enrichment of Base Excision Repair (BER) genes required for the repair of Reactive Oxygen Species (ROS) induced DNA damage (Fig. 1A) [14]. Thymine DNA glycosylase (TDG) removes thymine glycol, an oxidized thymine derivative [15]. Oxoguanine glycosylase 1 (OGG1) encodes the primary enzyme responsible for excision of 8-oxoguanine, the most common type of ROS induced DNA damage [16]. Poly-ADP ribose polymerase 1 (PARP1) catalyzes the covalent transfer of ADP-ribose moieties to a variety of nuclear proteins to initiate BER of oxidized nucleotides [17].

PARP1 inhibition recently emerged as a promising cancer therapy [18], [19], [20]. PARP1 inhibition exerts preferential cytotoxicity toward BRCA1/2 deficient tumors with defective homologous recombination (HR). Inactivation of BER by PARP1 inhibition leads to accumulation of DNA strand breaks that are toxic without repair by HR. Our results suggest that PARP1 inhibition additionally affords targeting of glioblastomas with hyperactive EGFR by disabling a mechanism required to counteract the deleterious effects of ROS. To test this hypothesis, we took the two independent siRNAs from the Qiagen library that are directed against PARP1 and confirmed their EGFRvIII specific radiosensitizing effects (Fig. S1). To further exclude the possibility of “off-target” effects associated with RNAi, we recapitulated this result using a siRNA distinct from the Qiagen library siRNA (Fig. 1B) and two pharmacologic PARP1 inhibitors, 3-aminobenzamide (3-AB) and NU1025 (Fig. 1C). Without radiation, PARP1 silencing or inhibition appeared to exert a mild toxic effect against U87MG-EGFRvIII cells that was not seen with U87MG cells. This effect was more pronounced for NU1025 in comparison to 3-AB. Since this effect was also observed using a PARP1 siRNA (Fig. 1B), the results suggest EGFR hyperactivation generates a cell state with increased dependency on PARP1 related function, in the absence of exogenous DNA damage. This dependency was magnified when combined with radiation. When combined with 2 Gy IR, the siRNA caused an approximately three-fold decrease in the clonogenic survival of the U87MG-EGFRvIII cells relative to the parental U87MG cells (p<0.05 by Student's t-test). Similar effects were seen using NU1025 and 3-AB though the magnitude of the effect (approximately two-fold) was slightly less than that seen with the siRNA. Though these effects were modest, they were consistently observed and statistically significant (p<0.05 by Student's t-test) (Fig. 1B, C). These results were reproduced using the U373MG glioblastoma line containing a tet-repressible EGFRvIII construct (Fig. 1D).

### EGFRvIII over-expressing glioblastoma cells exhibited increased levels of ROS, DNA damage, and genomic instability

**Figure 2:**
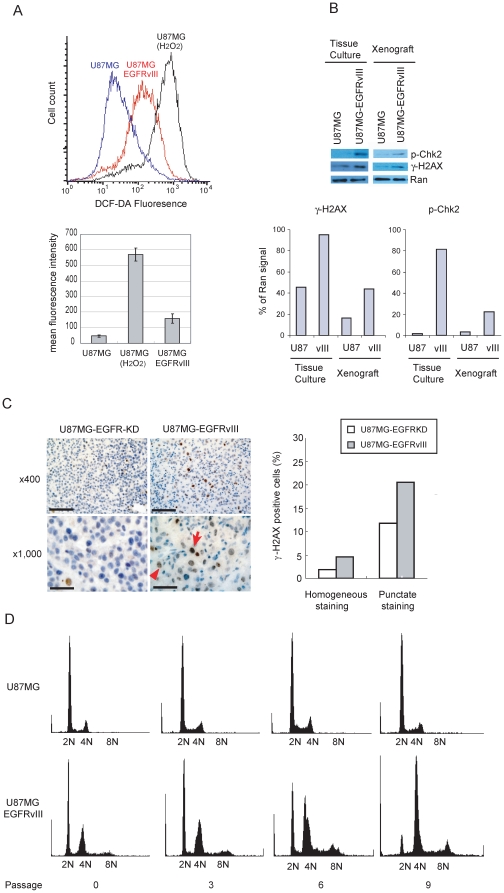
EGFR hyperactivation increases oxidative stress and DNA damage accumulation in glioma cells. (A) EGFRvIII expression is associated with increased DCF-DA fluorescence. A representative experiment (top). The mean and standard deviation of DCF-DA fluorescence derived from three independent experiments (bottom). (B) EGFRvIII expression is associated with increased γ-H2AX and p-Chk2 accumulation in tissue culture (T.C.) and xenograft models. Intensities of γ-H2AX and p-Chk2 bands were quantified and normalized to the intensity of the RAN loading control (bottom). The experiment was repeated three times with consistent results. A representative experiment is shown. (C) IHC of mouse xenografts confirmed that EGFRvIII expressing U87MG cells exhibit increased γ-H2AX accumulation. Scale bar, 100 µm (×400), 40 µm (×1,000). Percent of cells with γ-H2AX homogeneous (large arrow) or punctate nuclear staining (thin arrow, left panel) were quantified (right panel). (D) EGFRvIII expression in U87MG is associated with progressive ploidy alterations.

The increased reliance on BER in EGFRvIII over-expressing cells suggests elevated ROS levels in these cells. To test this hypothesis, U87MG and U87MG-EGFRvIII lines were assayed by the ROS-sensitive fluorophore DCF-DA[10]. U87MG-EGFRvIII cells exhibited markedly increased DCF-DA fluorescence, suggesting elevated ROS accumulation (Fig. 2A). To exclude DCF-DA dye related artifacts, the results were reproduced using two other assays for ROS including 8-oxoguanine and dihydroethidium fluorescence (Fig. S2). The increased DCF-DA fluorescence in the U87MG-EGFRvIII cell line was abolished by treatment with the EGFR inhibitor Erlotinib or EGFR siRNA (Fig. S3).

Unrepaired ROS induced DNA damages are often converted to DNA double-strand breaks (DSBs) [21]. We thus determined whether the increased ROS in U87MG-EGFRvIII cells culminate in DSB accumulation [12]. We found increased levels of γ-H2AX and phospho-Chk2, two biomarkers for DSB accumulation [22], in the U87MG-EGFRvIII line relative to the parental U87MG cells (Fig. 2B). A 2–3 fold increase in the levels of γ-H2AX and p-CHK2 were also seen in xenograft tumors derived from U87MG-EGFRvIII cells relative to those derived from U87MG, confirming our results *in vivo*. We further confirmed our observation by γ-H2AX immunohistochemical staining of tumors derived from U87MG-EGFRvIII and U87MG-EGFRKD (EGFRvIII-Kinase Dead) cells. The U87MG-EGFRKD cells express a mutant derivative of U87MG-EGFRvIII where the kinase activity was inactivated by a point mutation in the ATP-binding site [23]. Consistently, the U87MG-EGFRvIII tumors harbored increased number of cells with nuclear γ-H2AX staining relative to the U87MG-EGFRKD tumors (Fig. 2C) [24]. To ensure that these observations were not unique to the U87MG cells, we recapitulated these results using an U373MG line harboring a tet-repressible EGFRvIII construct (Fig. 1D).

DNA damage and strand breaks induced by excessive ROS accumulation are harbingers of genomic instability [21], [25]. Thus, we sought to determine whether EGFRvIII induced ROS predisposes accumulation of chromosomal aberrations. Sorted diploid U87MG and U87MG-EGFRvIII were serially passaged. The U87MG cells remained diploid after nine passages. However, the U87MG-EGFRvIII cells exhibited progressive ploidy changes (see increased >4N staining cells, Fig. 2D). Together, our results suggest that EGFR hyper-activation causes increased ROS accumulation, with resultant genomic instability.

### EGFR over-expression correlated with ROS accumulation and sensitivity to PARP1 inhibition

**Figure 3:**
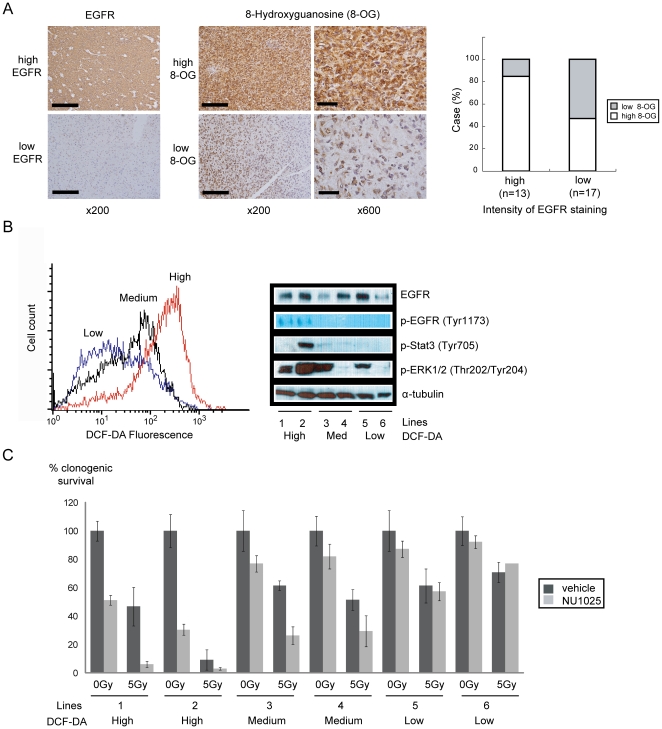
Clinical correlation between EGFR expression, oxidative stress and sensitivity to PARP inhibition. (A) EGFR expression correlated with 8-hydroxyguanosine (8-OG) staining in a glioblastoma microarray. Glioblastomas were stratified into high or low EGFR staining groups. Within each group, the percent of cases with high 8-OG staining is shown in white; low 8-OG staining in gray (right). Representative pictures of high and low staining patterns of EGFR and 8-OG are shown (left). Scale bar, 200 mm (×200), 50 mm (×600). (B) Correlation between DCF-DA fluorescence and EGFR. Primary glioblastoma lines were grouped based on DCF-DA staining (left). p-EGFR, p-Stat3, and p-ERK1/2 levels were assessed (right). (C) the level of oxidative stress/EGFR activation correlated with sensitivity to PARP1 inhibition.

We wished to confirm our findings in clinical specimens by assessing whether high 8-hydroxyguanosine (8-OG) IHC staining, a well known marker for ROS levels [26], correlates with EGFR immunostaining. Using a commercially available glioblastoma microarray (US Biomax, Rockville, MD), we showed that EGFR staining tends to parallel 8-OG staining (i.e. high EGFR staining glioblastomas tend to exhibit high 8-OG staining; Fig. 3A, p<0.05). The same correlation was seen in low-grade gliomas (Fig. S5).

We further tested the correlation between EGFR hyperactivity, ROS, and sensitivity to PARP1 using a panel of primary glioblastoma cell lines. These lines were classified by DCF-DA fluorescence intensity (Fig. 3A). The two lines with the highest DCF-DA fluorescence exhibited the highest degree of EGFR activation, as measured by levels of phospho-EGFR (Tyr 1173), phospho-Stat3, and phospho-ERK1/2 [27] (Fig. 3B). Interestingly, these lines were sensitive to PARP1 inhibition without IR. The two lines with moderate DCF-DA fluorescence exhibited IR sensitization upon PARP1 inhibition. Such sensitization was not observed in the lines with low DCF-DA fluorescence (Fig. 3C). RT-PCR of the EGFR transcript from the two high EGFR/DCF-DA lines revealed that neither harbored the EGFRvIII variant (Fig. S6). Together, these results suggest that EGFR hyper-activation in the absence of EGFRvIII is sufficient to induce ROS accumulation and sensitivity to PARP1 inhibition.

### BER gene expression inversely correlated with survival in patients afflicted with glioblastoma with high EGFR expression

**Figure 4:**
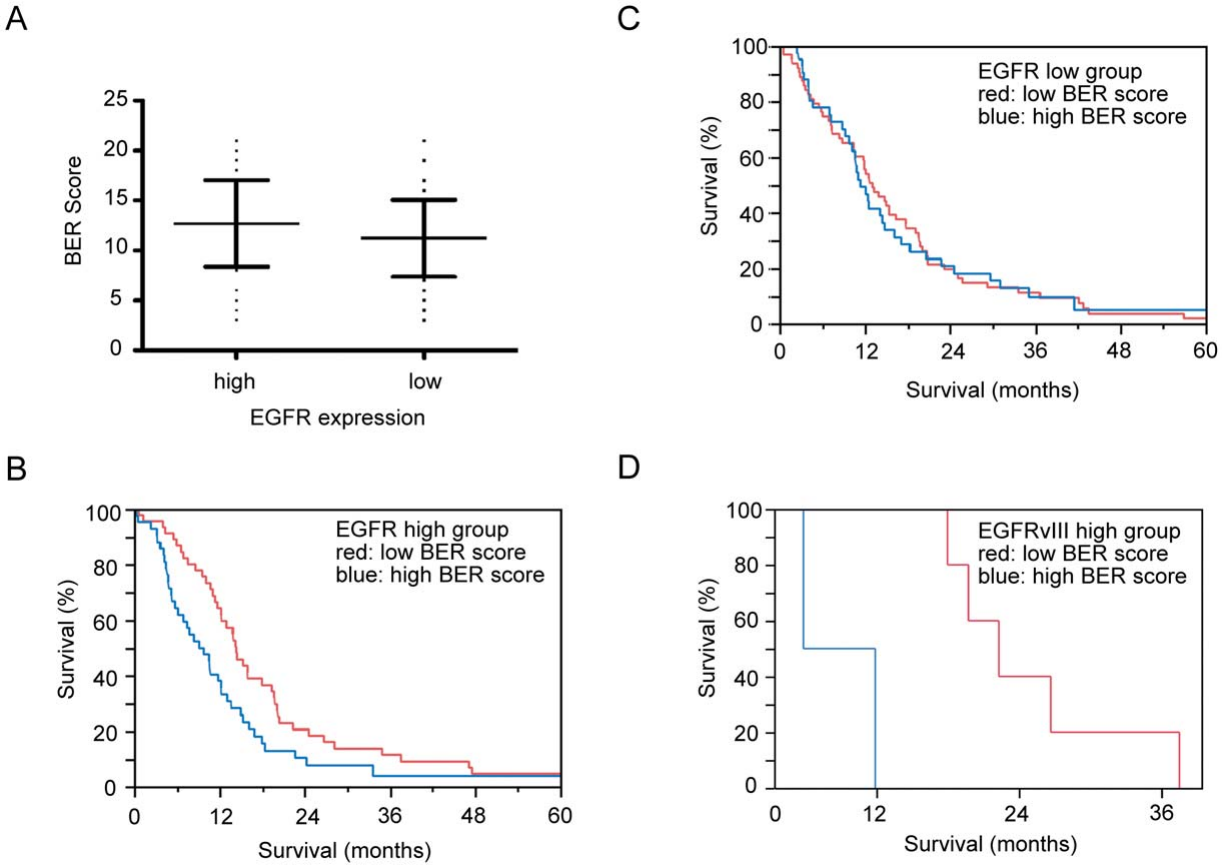
Association between EGFR and BER gene expression and clinical outcome. (A) In clinical specimens, EGFR expression and BER score showed no correlation. (B) Patients with high EGFR expressing glioblastomas with low BER score exhibited improved survival, log rank p = 0.01. Red: low BER score; blue: high BER score; median survival times of 14.5 and 9.2 months, respectively. (C) BER gene expression did not impact patients with glioblastomas exhibiting low EGFR expression, log rank p = 0.99. (D) Patients with EGFRvIII expressing glioblastomas with low BER score exhibited improved survival, log rank p = 0.008. Red: low BER score; blue: high BER score; median survival times of 22.6 and 8.9 months, respectively.

Since BER activity can be transcriptionally regulated [28], we wished to determine whether EGFR hyperactive glioblastomas increased transcription of BER genes in response to increased oxidative stress. Using The Cancer Genome Atlas (TCGA) glioblastoma database [1], [29], tumors were stratified into groups based on EGFR transcript level. Because most of the BER genes functionally overlap [12], [28], we developed a BER score (see Methods) to assess global BER transcription as a proxy for overall BER activity. The BER score did not appear to correlate with EGFR transcript level (Fig. 4A). Given these findings, we reasoned that tumors with high EGFR expression (high ROS) and low BER gene expression (diminished compensatory mechanism) should exhibit reduced survival fitness and therefore associate with favorable patient survival. Indeed, patients with glioblastomas exhibiting high EGFR expression and low BER score exhibited improved survival (Fig. 4B). For the patient group with high EGFR expressing glioblastomas, median survival times with low and high BER scores were 14.5 and 9.2 months, respectively (log rank p = 0.01). Strikingly, such a survival benefit was not observed in the low EGFR group (log rank p = 0.99, Fig. 4C). Among the seven TCGA patients with EGFRvIII expressing tumors, median survival times with low and high BER scores were 22.6 and 8.9 months, respectively (log rank p = 0.008, Fig. 4D), further supporting the importance of BER in EGFR hyperactive glioblastomas.

Our results indicate that ROS and DSB accumulation in EGFR hyperactive glioblastoma cells is mitigated by BER and lay the conceptual framework for the application of PARP1/BER inhibition beyond the subset of tumors deficient in HR [18], [19], [20]. To the extent that BER genes have yet been implicated in glioblastoma oncogenesis, the framework constitutes a form of “non-oncogene” addiction. This addiction occurs in the absence of exogenous DNA damage. That is, the EGFR hyperactive glioblastomas accumulated increased ROS/DSBs and were hypersensitive to BER inhibition at baseline. IR induced ROS/DSBs magnified this form of non-oncogene addiction by “overloading” the already taxed BER pathway. The efficacy of IR in glioblastoma patients can, in part, be rationalized by this paradigm. A corollary of this paradigm is that therapeutic insights can be derived by mapping the intersection between the various forms of non-oncogene addiction and the molecular effects of existing glioblastoma therapies.

The work further provides several insights into the clinical translation of the “non-oncogene addiction” framework. First and foremost, the effects of therapies designed based on the principles of “oncogene addiction” and of “non-oncogene addiction” are inherently antagonistic. In our study, EGFR inhibition leads to reduction of ROS, obviating the need for BER. In this context, combination of PARP1 and EGFR inhibition would not be desirable. Additionally, there appears to be sufficient variability in the pathways of oncogenesis [30], such that not every EGFR hyperactive glioblastoma develops increased BER capacity. The discrepancy between oncogenic stress and stress support pathways in select tumors offers an opportunity to maximize therapeutic efficacy. We propose the use of the transcriptome-based BER score and EGFR status as a means of capitalizing on this opportunity in clinical trial design involving BER inhibitors. Finally, our screen results suggest that a much larger set of non-oncogenes serves to support EGFRvIII induced oncogenic stress. Indeed, EGFR activation has been shown to modulate potential oncogenic stress support pathways including DSB repair [31] and apoptosis [32]. Understanding the physiologic interactions between the various stress support pathways should afford the development of synergistic drug combinations [4] ultimately required for meaningful therapeutic efficacy.

## Materials and Methods

### Cell culture and reagents

The U87MG, U87MG-EGFRvIII, U87MG-EGFRKD, and U373MG tet-EGFRvIII cell lines were obtained from Dr. Webster K. Cavenee and propagated as reported [6], [23]. Primary glioblastoma cell lines were derived from fresh surgical specimens after obtaining written informed consent under Institutional Review Board-approved Dana-Farber/Harvard Cancer Center Protocol 07-231. These were passaged as described for U87MG; passage 2–3 lines were used. NU1025 (Sigma Aldrich, St. Louis, MO), 3-aminobenzamide (3-AB, Sigma), 4-amino-1,8-naphthalimide (4-ANI, Calbiochem, Gibbstown, NJ) and doxycycline (Sigma) were dissolved in DMSO. N-acetylcysteine (NAC, Sigma) and hydrogen peroxide (H_2_O_2_, Sigma) were dissolved in culture media.

### Reactive Oxygen Species (ROS) and chromosomal instability assays

Levels of ROS in U87MG and U87MG-EGFRvIII cells were assessed using the OxyDNA Assay Kit (Calbiochem), DCF-DA (Invitrogen, Carlsbad, CA), and dihydroethidium (Invitrogen) according to manufacturers' instructions. To assay chromosomal instability, sorted diploid populations of U87MG and U87MG-EGFRvIII were passaged every 3 days, and cell cycle distributions were analyzed by flow cytometry after propidium iodide staining [33].

### siRNA library screen and validation

Cells were transfected with 20 nM of siRNA oligonucleotides using HiPerfect transfection reagent (QIAGEN, Valencia, CA). Twenty-four hours after transfection, cells were irradiated with 5 Gy IR. Viability was assessed 72 hours after irradiation using the CellTiter-Glo Luminescent Cell Viability Assay kit (Promega, Madison, WI). The experiment was performed twice for each cell line on independent days to allow for statistical analysis. The corrected viability for cells transfected with each siRNA was calculated as a percentage of the mean viability of the control wells containing GFP or scrambled siRNAs (Qiagen DNA repair subset v2.0) for each 96-well plate. The corrected viability of the irradiated cells was then subtracted from the corrected viability of the non-irradiated cells to calculate the relative viability after irradiation for each respective gene target. The mean of these values (referred to as the mean percent viability after irradiation) was calculated (averaging the two distinct siRNAs directed against the same target gene in each of the two experiments) and termed the radiation effect. The radiation effect derived from the U87MG-EGFRvIII line was then subtracted from that derived from the U87MG line. This value was expressed as a percent of the radiation effect for EGFRvIII and reported as the EGFRvIII IR index. The siRNA targets are ranked based on this index [33].

### Immunoblotting and immunohistochemistry

Western blotting and IHC were performed using standard techniques as previously described [6], [33]; see Methods S1 for details.

### siRNA transfection

The initial siRNA screen was performed using the Qiagen DNA repair subset v2.0 siRNA library. Subsequent PARP1 confirmation studies were performed using an independent siRNA directed against PARP1 (ON-TARGET plus J-006656-05-0005, Dharmacon, Denver CO). Qiagen Hs_EGFR_12 (5′-CAGGAACTGGATATTCTGAAA-3′) was used for EGFR silencing. The control siRNA was obtained from Qiagen (AllStars Negative Control and anti-GFP: 5′-AACACTTGTCACTACTTTCTC-3′). The siRNA transfections were done by using either RNAiMax (Invitrogen) or HiPerfect reagent (Qiagen). In brief, 1×10^5^ cells were plated on 6-well plates the day before transfection, then 20 nM of siRNA was transfected according to manufacturer's protocol for 24 hours prior to subsequent manipulations.

### Viability assays

Cells were seeded into 10 cm plates and treated for 24 hours with various siRNAs or PARP inhibitors, and then irradiated with ionizing radiation (IR). Cells were trypsinized 24 hours after irradiation and plated in serial dilution. 10–14 days after irradiation, cells were fixed and stained with crystal violet (0.1%) and numbers of colonies were counted. All experiments were performed in triplicates and repeated at least twice. For doxycycline experiments, cells were treated with doxycycline for 72 hours prior to subsequent manipulation.

### RT-PCR analysis of EGFRvIII

Total RNA was isolated from primary glioblastoma cultures, U87MG and U87MG-EGFRvIII cell lines (2×10^6^ cells) using the RNeasy Mini kit (Qiagen, Valencia, CA). Standard RT-PCR was done with primers designed to flank the deletion of exons 2 to 7 (5′-ATGCGACCCTCCGGGACG-3′ and 5′-ATTCCGTTACACACTTTGCGGC-3′; final reaction concentration = 100 nM). Reverse transcription was done at 50°C for 30 minutes followed by enzyme inactivation at 95°C for 15 minutes. This was followed by 35 cycles of (94°C: 1 min; 55°C: 1 min; 72°C: 1 min) and a final extension of 72°C for 10 minutes. PCR products were visualized on a 1% agarose gel containing ethidium bromide.

### Biostatistical analysis

Glioblastoma gene expression and correlating clinical data were obtained from The Cancer Genome Atlas (http://www.broadinstitute.org/~gadgetz/TCGA/TCGA_206_median.txt) [1], [29]. Tumors were categorized as showing high (greater than mean) vs. low EGFR expression. An exhaustive literature search revealed 24 genes implicated in BER (PARP1, PARP2, XRCC1, LIG3, POLB, UNG, SMUG1, MBD4, TDG, DUT, OGG1, NTHL1, NEIL1, NEIL2, NEIL3, NUDT1, MUTYH, APEX1, APEX2, PNKP, MGMT, ALKBH2, ALKBH3 and MPG) [12]. For each tumor, one point was added for each BER gene showing greater than mean expression. The sum of these points was termed the BER score. A high BER score was defined as one greater than 12. Kaplan-Meier plots were generated using JMP (SAS Institute, Cary, NC). Seven tumors were previously identified via SNP array-based copy number analysis as expressing the EGFRvIII mutation; these showed a loss of exons 2–7 relative to downstream EGFR exons [1], [29].

## Supporting Information

Methods S1 Supplemental Materials and Methods.
(0.05 MB DOC)

Figure S1 PARP1 silencing preferentially radiosensitized EGFRvIII hyperactive glioblastoma cells. Two independent siRNAs against PARP1 preferentially sensitized U87MG-EGFRvIII cells to IR (left). The efficency of knock down is shown in the right column. The siRNAs were taken from the Qiagen DNA repair subset v2.0. Cells were transfected with 20 nM of siRNA oligonucleotides using HiPerfect transfection reagent (QIAGEN, Valencia, CA). Twenty-four hours after transfection, cells were irradiated with 2 Gy IR. Viability was assessed by clonogenic survival as described in methods. In parallel, cells were seeded into a 10 cm plate and treated as described above. RNA extraction was performed at 72 hours after irradiation. The Qiagen QuantiTect SYBR Green RT-PCT Kit was used to quantify the efficiency of gene silencing as per manufacturer's instructions. Each experiment was repeated twice. Results from a representative experiment is shown.
(0.12 MB TIF)

Figure S2 EGFRvIII over-expression induced increased ROS accumulation. (A) Levels of 8-oxoguanine, the most common form of ROS induced DNA damage, are increased in U87MG-EGFRvIII cells relative to U87MG cells. U87MG and U87MG-EGFRvIII cells were harvested, washed with PBS, and fixed with ice-cold 70% ethanol. The cells were then washed with PBS and incubated with FITC-conjugated 8-oxoguanine probe (1∶100) for 60 min. Fluorescence was measured by FACS. (B) EGFRvIII over-expression in U87MG is associated with increased ROS accumulation as gauged by dihydroethidium fluorescence. U87MG and U87MG-EGFRvIII cells were harvested, washed with PBS, and incubated with dihydroethidium (5 mM) for 15 min. As a positive control, U87MG cells were also treated with 0.03% hydrogen peroxide (H2O2) for 30 min before harvest. Levels of dihydroethidium fluorescence were measured by FACS. Each bar depicts mean dihydroethidium fluorescence intensity ± SEM (normalized to unstained control) derived from triplicates.
(0.47 MB TIF)

Figure S3 EGFR inhibition or silencing decreased ROS level in U87MG-EGFRvIII cells. (A) EGFR inhibition by erlotnib in U87MG-EGFRvIII cells decreased ROS levels. U87MG-EGFRvIII cells were treated with DMSO or erlotinib (10 mM) or N-acetylcysteine (NAC, Sigma) as a positive control for 48 hours. Cells were harvested, washed with PBS, and incubated with DCF-DA (5 mM) for 15 min. Cells were then washed with PBS and analyzed by FACS. (B) EGFR silencing reduces ROS levels in U87MG-EGFRvIII cells. U87MG-EGFRvIII cells were transfected with siRNA against EGFR or a control siRNA. Cells were cultured for another 72 hours. DCF-DA fluorescence was measured by FACS (left panel). Efficiency of EGFRvIII silencing is shown in the right panel.
(0.48 MB TIF)

Figure S4 EGFRvIII expression is associated with DNA damage accumulation in U373MG cells. U373MG cells harboring a tet-repressible EGFRvIII construct [1] were treated with doxycycline (1 mM) or vehicle for 72 hours. Cell lysates were prepared and levels of γ-H2AX and p-Chk2 were analyzed (top panel). Intensity of γ-H2AX and p-Chk2 bands were quantified and normalized to the intensity of the RAN loading control (bar graph in the bottom panel).
(0.38 MB TIF)

Figure S5 EGFR expression correlates with 8-hydroxyguanosine (8-OG) staining in low-grade gliomas. A low-grade gliomas microarray (US Biomax) was stained for EGFR and 8-OG (see Supplemental Materials and Methods). Samples were stratified into high or low EGFR staining groups. Within each group, the percent of cases with high 8-OG staining is shown in white; low 8-OG staining cases shown in gray. High EGFR staining glioblastomas tend to exhibit high 8-OG staining (p<0.05 by Student's t-test).
(0.18 MB TIF)

Figure S6 Primary glioblastoma lines used did not harbor EGFRvIII transcript. EGFR transcripts in primary glioblastoma lines were examined by RT-PCR. Ethidium bromide stained gel showing EGFR PCR products from two primary glioblastoma lines, GBM1 and GBM2. mRNAs isolated from U87MG (expressing EGFR) and U87MG-EGFRvIII were used as controls.
(0.31 MB TIF)

## References

[1] Cancer Genome Atlas Research N (2008). Comprehensive genomic characterization defines human glioblastoma genes and core pathways.. Nature.

[2] Parsons DW, Jones S, Zhang X, Lin JC, Leary RJ (2008). An integrated genomic analysis of human glioblastoma multiforme.. Science.

[3] Mellinghoff IK, Wang MY, Vivanco I, Haas-Kogan DA, Zhu S (2005). Molecular determinants of the response of glioblastomas to EGFR kinase inhibitors.. New England Journal of Medicine.

[4] Luo J, Solimini NL, Elledge SJ (2009). Principles of cancer therapy: oncogene and non-oncogene addiction.. Cell.

[5] Luo J, Emanuele MJ, Li D, Creighton CJ, Schlabach MR (2009). A genome-wide RNAi screen identifies multiple synthetic lethal interactions with the Ras oncogene.. Cell.

[6] Nishikawa R, Ji XD, Harmon RC, Lazar CS, Gill GN (1994). A mutant epidermal growth factor receptor common in human glioma confers enhanced tumorigenicity.. Proc Natl Acad Sci U S A.

[7] Weinstein IB (2002). Addiction to oncogenes–the Achilles heal of cancer.. Science.

[8] Stommel JM, Kimmelman AC, Ying H, Nabioullin R, Ponugoti AH (2007). Coactivation of receptor tyrosine kinases affects the response of tumor cells to targeted therapies.. Science.

[9] Lo HW, Hsu SC, Ali-Seyed M, Gunduz M, Xia W (2005). Nuclear interaction of EGFR and STAT3 in the activation of the iNOS/NO pathway.. Cancer Cell.

[10] Lee AC, Fenster BE, Ito H, Takeda K, Bae NS (1999). Ras proteins induce senescence by altering the intracellular levels of reactive oxygen species.. J Biol Chem.

[11] Denko NC, Giaccia AJ, Stringer JR, Stambrook PJ (1994). The human Ha-ras oncogene induces genomic instability in murine fibroblasts within one cell cycle.. Proc Natl Acad Sci U S A.

[12] Friedberg E, Walker G, Siede W, Wood R, Schultz R (2006). DNA repair and mutagenesis.

[13] Jackson SP, Bartek J (2009). The DNA-damage response in human biology and disease.. Nature.

[14] Helbock HJ, Beckman KB, Ames BN (1999). 8-Hydroxydeoxyguanosine and 8-hydroxyguanine as biomarkers of oxidative DNA damage.. Methods Enzymol.

[15] Yoon JH, Iwai S, O'Connor TR, Pfeifer GP (2003). Human thymine DNA glycosylase (TDG) and methyl-CpG-binding protein 4 (MBD4) excise thymine glycol (Tg) from a Tg:G mispair.. Nucleic Acids Res.

[16] Hazra TK, Das A, Das S, Choudhury S, Kow YW (2007). Oxidative DNA damage repair in mammalian cells: a new perspective.. DNA Repair (Amst).

[17] Haince JF, Rouleau M, Hendzel MJ, Masson JY, Poirier GG (2005). Targeting poly(ADP-ribosyl)ation: a promising approach in cancer therapy.. Trends Mol Med.

[18] Fong PC, Boss DS, Yap TA, Tutt A, Wu P (2009). Inhibition of poly(ADP-ribose) polymerase in tumors from BRCA mutation carriers.. N Engl J Med.

[19] Farmer H, McCabe N, Lord CJ, Tutt AN, Johnson DA (2005). Targeting the DNA repair defect in BRCA mutant cells as a therapeutic strategy.. Nature.

[20] Bryant HE, Schultz N, Thomas HD, Parker KM, Flower D (2005). Specific killing of BRCA2-deficient tumours with inhibitors of poly(ADP-ribose) polymerase.. Nature.

[21] Rassool FV, Gaymes TJ, Omidvar N, Brady N, Beurlet S (2007). Reactive oxygen species, DNA damage, and error-prone repair: a model for genomic instability with progression in myeloid leukemia?. Cancer Res.

[22] Harper JW, Elledge SJ (2007). The DNA damage response: ten years after.. Mol Cell.

[23] Huang HS, Nagane M, Klingbeil CK, Lin H, Nishikawa R (1997). The enhanced tumorigenic activity of a mutant epidermal growth factor receptor common in human cancers is mediated by threshold levels of constitutive tyrosine phosphorylation and unattenuated signaling.. Journal of Biological Chemistry.

[24] Bartkova J, Horejsi Z, Koed K, Kramer A, Tort F (2005). DNA damage response as a candidate anti-cancer barrier in early human tumorigenesis.. Nature.

[25] Sallmyr A, Fan J, Rassool FV (2008). Genomic instability in myeloid malignancies: increased reactive oxygen species (ROS), DNA double strand breaks (DSBs) and error-prone repair.. Cancer Lett.

[26] Kim HW, Murakami A, Williams MV, Ohigashi H (2003). Mutagenicity of reactive oxygen and nitrogen species as detected by co-culture of activated inflammatory leukocytes and AS52 cells.. Carcinogenesis.

[27] Ono M, Kuwano M (2006). Molecular mechanisms of epidermal growth factor receptor (EGFR) activation and response to gefitinib and other EGFR-targeting drugs.. Clin Cancer Res.

[28] Tudek B (2007). Base excision repair modulation as a risk factor for human cancers.. Mol Aspects Med.

[29] Verhaak R, Hoadley K, Purdom E, Wang V, Qi Y (2010). Integrated Genomic Analysis Identifies Clinically Relevant Subtypes of Glioblastoma Characterized by Abnormalities in *PDGFRA*, *IDH1*, *EGFR*, and *NF1* p98.. Cancer Cell.

[30] Bild AH, Yao G, Chang JT, Wang Q, Potti A (2006). Oncogenic pathway signatures in human cancers as a guide to targeted therapies.. Nature.

[31] Mukherjee B, McEllin B, Camacho CV, Tomimatsu N, Sirasanagandala S (2009). EGFRvIII and DNA double-strand break repair: a molecular mechanism for radioresistance in glioblastoma.. Cancer Res.

[32] Nagane M, Levitzki A, Gazit A, Cavenee WK, Huang HJ (1998). Drug resistance of human glioblastoma cells conferred by a tumor-specific mutant epidermal growth factor receptor through modulation of Bcl-XL and caspase-3-like proteases.. Proc Natl Acad Sci U S A.

[33] Chen CC, Kennedy RD, Sidi S, Look AT, D'Andrea A (2009). CHK1 inhibition as a strategy for targeting Fanconi Anemia (FA) DNA repair pathway deficient tumors.. Mol Cancer.

