# Correction: Targeting EGFR Induced Oxidative Stress by PARP1 Inhibition in Glioblastoma Therapy

**DOI:** 10.1371/annotation/9b9cc4ae-ad56-464f-ab97-59f096b5c0eb

**Published:** 2011-04-27

**Authors:** Masayuki Nitta, David Kozono, Richard Kennedy, Jayne Stommel, Kimberly Ng, Pascal O. Zinn, Deepa Kushwaha, Santosh Kesari, Frank Furnari, Katherine A. Hoadley, Lynda Chin, Ronald A. DePinho, Webster K. Cavenee, Alan D'Andrea, Clark C. Chen

The authors would like to add Maria-del-Mar Inda and Jill Wykosky to the byline. The updated byline is: Masayuki Nitta1#, David Kozono1,2#, Richard Kennedy3, Jayne Stommel4, Kimberly Ng1, Pascal O. Zinn1, Deepa Kushwaha1, Santosh Kesari5, Maria-del-Mar Inda6, Jill Wykosky6, Frank Furnari6, Katherine A. Hoadley7, Lynda Chin4, Ronald A. DePinho4, Webster K. Cavenee6, Alan D'Andrea1, Clark C. Chen1,8*

The two new authors should be added to the author contributions "Contributed reagents/materials/analysis tools" section. The section should read "Contributed reagents/materials/analysis tools: JS SK M-d-MI JW FBF LC RAD WKC ADD CCC."

