# Supplementary material for: Targeting EGFR Induced Oxidative Stress by PARP1 Inhibition in Glioblastoma Therapy
Source: PLoS One. 2010 May 24;5(5):e10767. doi: 10.1371/journal.pone.0010767 (PMC2879424; doi:10.1371/journal.pone.0010767)
Supplement: Methods S1 — Supplemental Materials and Methods. (0.05 MB DOC) [file pone.0010767.s001.doc]

**Methods S1**

**Immunoblotting and immunohistochemistry.** Antibodies used in this study are described in the following table:

|  | Antibody | Manufacturer | Catalog number | Dilution |
| --- | --- | --- | --- | --- |
| Western blot | Phospho-Chk2 (Thr68) | Cell Signaling | 2661 | 1:500 |
|  | γ-H2AX | Cell Signaling | 2577 | 1:500 |
|  | EGFR | Santa Cruz Biotechnology | 1005 | 1:500 |
|  | Phospho-EGFR (Tyr1173) | Cell Signaling | 4407 | 1:500 |
|  | Phospho-STAT3 (Tyr705) | Cell Signaling | 9138 | 1:500 |
|  | Phospho-ERK1/2 (Thr202/Tyr204) | Cell Signaling | 9106 | 1:500 |
|  | GAPDH | Abcam | Ab9484 | 1:3,000 |
|  | α-tubulin | Sigma-Aldrich | T9026 | 1:5,000 |
|  | RAN | BD Biosciences | 610340 | 1:20,000 |
| IHC | 8-hydroxyguanosine | Rockland | 21554 | 1:200 |
|  | EGFR | Santa Cruz Biotechnology | 1005 | 1:50 |
|  | γ-H2AX | Cell Signaling | 2577 | 1:50 |

For extract preparation from tissue cultured cells, cells were lysed using 1x sample buffer (50 mM Tris-HCl, pH 6.8, 86 mM 2-mercaptoethanol, 2% sodium dodecyl sulfate (SDS)), boiled for 10 minutes, and subjected to fractionation by SDS–PAGE. For extract preparation from xenograft tumor cells, subcutaneous tumors were surgically removed 1 month after injection (1 × 10^6^ cells injected) and stored in liquid nitrogen. A piece of frozen tumor was ground with mortar and pestle under liquid nitrogen. The ground powder was then resuspended in cell lysis buffer (#9803, Cell Signaling) and subjected to analysis as described below.

Transfer of proteins to a nitrocellulose membrane was achieved using a Bio-Rad (Richmond, CA) transfer apparatus. Membranes were then blocked for 60 min with 5% commercial non-fat dry milk or 1% BSA dissolved in TBS-T (Tris-buffered saline containing 0.1% Tween 20). After blocking, membranes were incubated with the various primary antibodies (see table) overnight, washed, and exposed to the appropriate secondary antibodies (GE Healthcare, Piscataway, NJ) at 1:5000 dilution in TBS-T. Detection was performed using an enhanced chemiluminescence system (Western Lightning Plus-ECL, PerkinElmer, Waltham MA). Image quantitation was done using the Scion image quantitation software (Scion Corporation, Fredrick, MD).

For the IHC studies, slides were incubated at 60˚C for 60 min in a hybridization oven to remove secondary paraffin layers. Antigen retrieval was accomplished with incubating the sections for 10 minutes in citrate buffer at sub-boiling temperature. Primary antibodies were applied and incubated over night at 4˚C. The reaction was visualized by DAB (Vector Laboratories, Burlingame, CA). The sections were counterstained with Mayer’s hematoxylin and mounted with Permount™ Mounting Medium (Electron Microscopy Sciences, Hatfield, PA).

Staining of EGFR was classified as 0, 1+, 2+ or 3+ per convention [1,2]; tumors were stratified as showing low expression if between 0 and 2+ or as high expression if 3+. Staining of 8-OG was classified as low if nuclear staining was present in less than 50% of cells and cytoplasmic staining was low/undetectable, high if nuclear staining was present in more than 50% and cells or if there was strong cytoplasmic staining. Grading was performed by three independent observers with training in pathology. Statistical comparisons were performed using the non-parametric Mann-Whitney test.

**REFERENCES**

1. Atkins D, Reiffen KA, Tegtmeier CL,Winther H, Bonato MS, Storkel S. (2004) Immunohistochemical detection of EGFR in paraffin-embedded tumor tissues: variation in staining intensity due to choice of fixative and storage time of tissue sections. J Histochem Cytochem 52: 893-901.

2. Gamboa-Dominguez A, Dominguez-Fonseca C, Quintanilla-Martinez L, Reyes-Gutierrez E, Green D, et al. (2004) Epidermal growth factor receptor expression correlates with poor survival in gastric adenocarcinoma from Mexican patients: a multivariate analysis using a standardized immunohistochemical detection system. Modern Pathol 17: 579-587.
