# Supplementary figures and images for: Targeting EGFR Induced Oxidative Stress by PARP1 Inhibition in Glioblastoma Therapy
Source: PLoS One. 2010 May 24;5(5):e10767. doi: 10.1371/journal.pone.0010767 (PMC2879424; doi:10.1371/journal.pone.0010767)

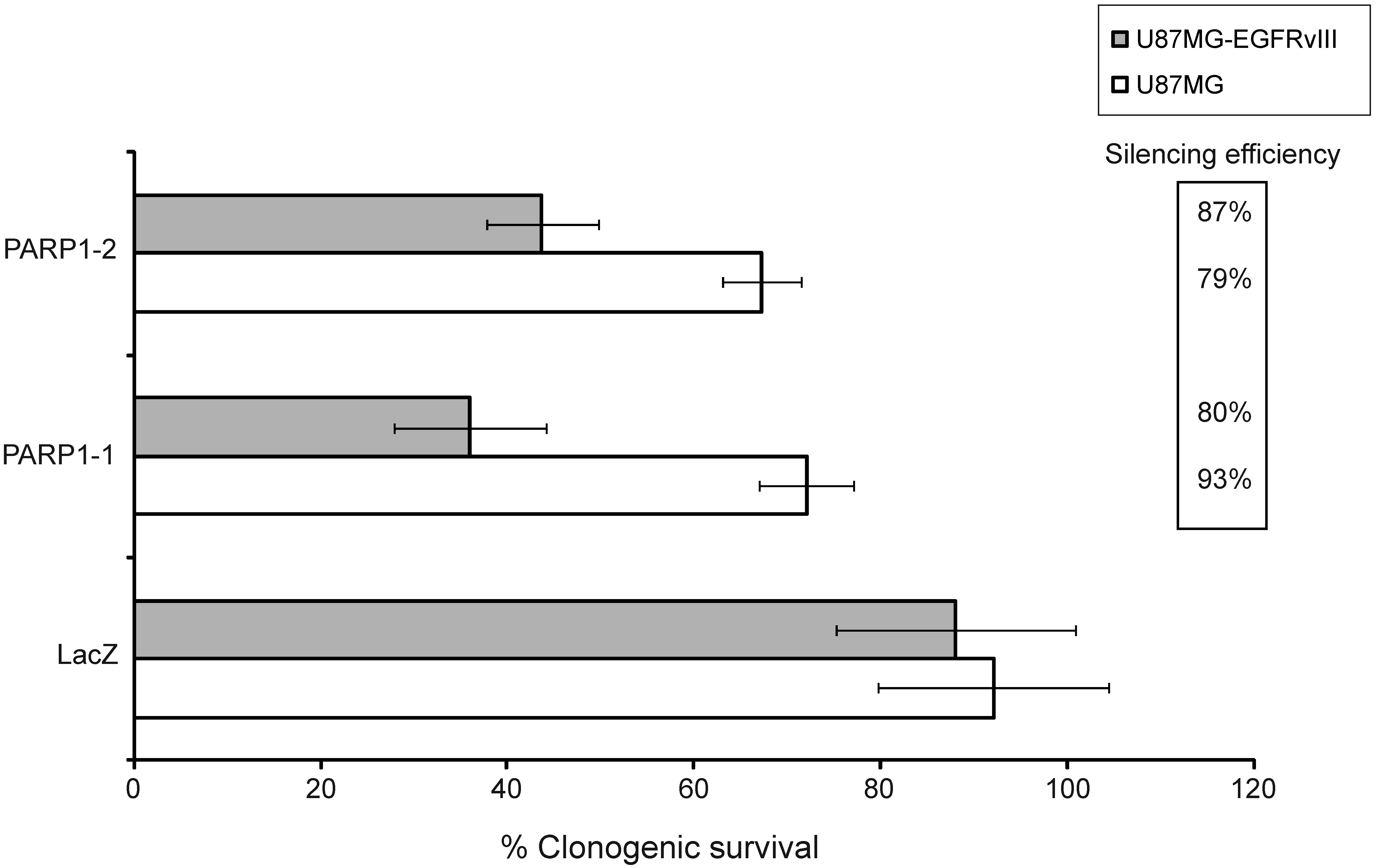

Supplement: Figure S1 — PARP1 silencing preferentially radiosensitized EGFRvIII hyperactive glioblastoma cells. Two independent siRNAs against PARP1 preferentially sensitized U87MG-EGFRvIII cells to IR (left). The efficency of knock down is shown in the right column. The siRNAs were taken from the Qiagen DNA repair subset v2.0. Cells were transfected with 20 nM of siRNA oligonucleotides using HiPerfect transfection reagent (QIAGEN, Valencia, CA). Twenty-four hours after transfection, cells were irradiated with 2 Gy IR. Viability was assessed by clonogenic survival as described in methods. In parallel, cells were seeded into a 10 cm plate and treated as described above. RNA extraction was performed at 72 hours after irradiation. The Qiagen QuantiTect SYBR Green RT-PCT Kit was used to quantify the efficiency of gene silencing as per manufacturer's instructions. Each experiment was repeated twice. Results from a representative experiment is shown. (0.12 MB TIF) [file pone.0010767.s002.tif]

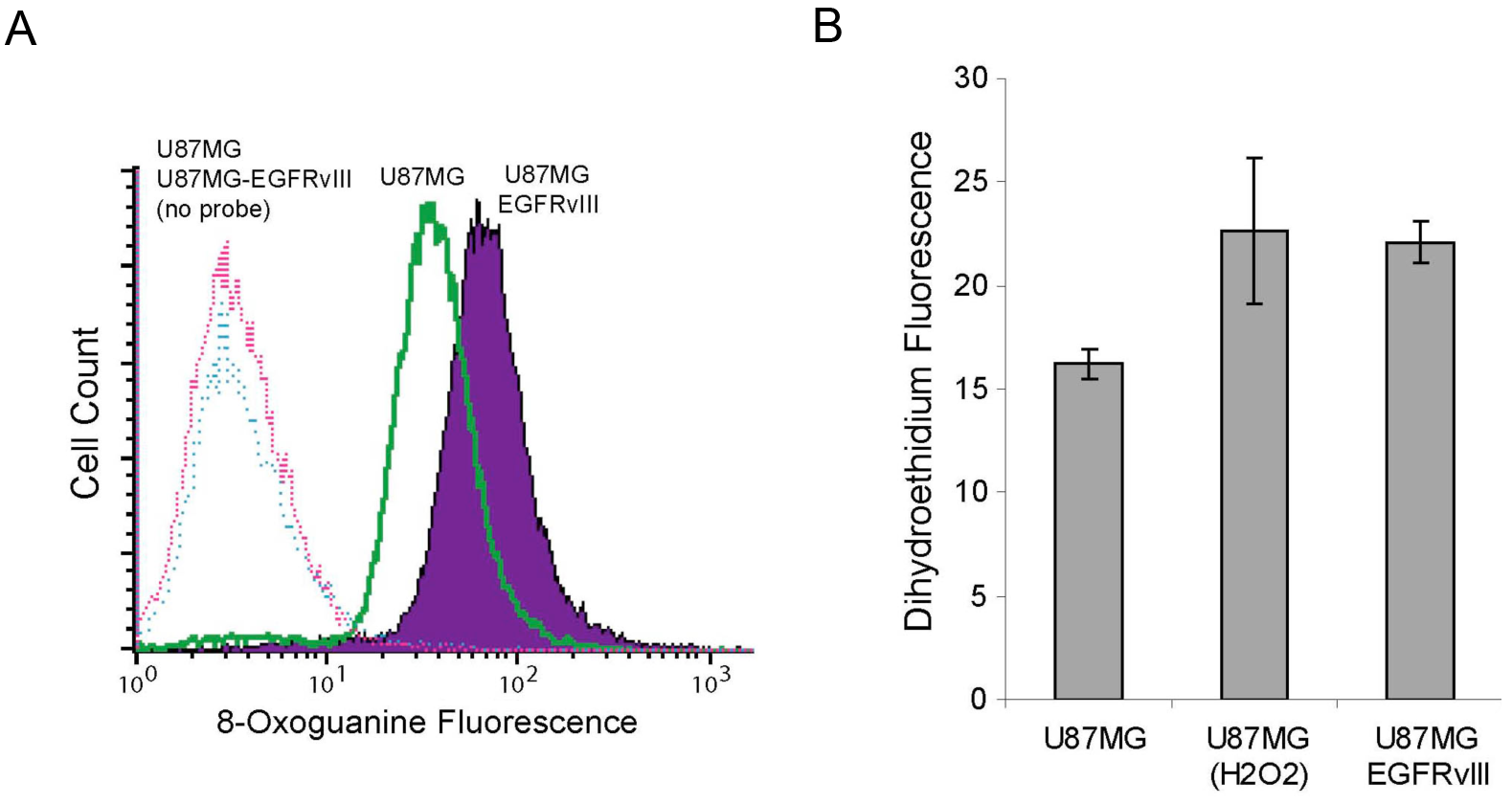

Supplement: Figure S2 — EGFRvIII over-expression induced increased ROS accumulation. (A) Levels of 8-oxoguanine, the most common form of ROS induced DNA damage, are increased in U87MG-EGFRvIII cells relative to U87MG cells. U87MG and U87MG-EGFRvIII cells were harvested, washed with PBS, and fixed with ice-cold 70% ethanol. The cells were then washed with PBS and incubated with FITC-conjugated 8-oxoguanine probe (1∶100) for 60 min. Fluorescence was measured by FACS. (B) EGFRvIII over-expression in U87MG is associated with increased ROS accumulation as gauged by dihydroethidium fluorescence. U87MG and U87MG-EGFRvIII cells were harvested, washed with PBS, and incubated with dihydroethidium (5 mM) for 15 min. As a positive control, U87MG cells were also treated with 0.03% hydrogen peroxide (H2O2) for 30 min before harvest. Levels of dihydroethidium fluorescence were measured by FACS. Each bar depicts mean dihydroethidium fluorescence intensity ± SEM (normalized to unstained control) derived from triplicates. (0.47 MB TIF) [file pone.0010767.s003.tif]

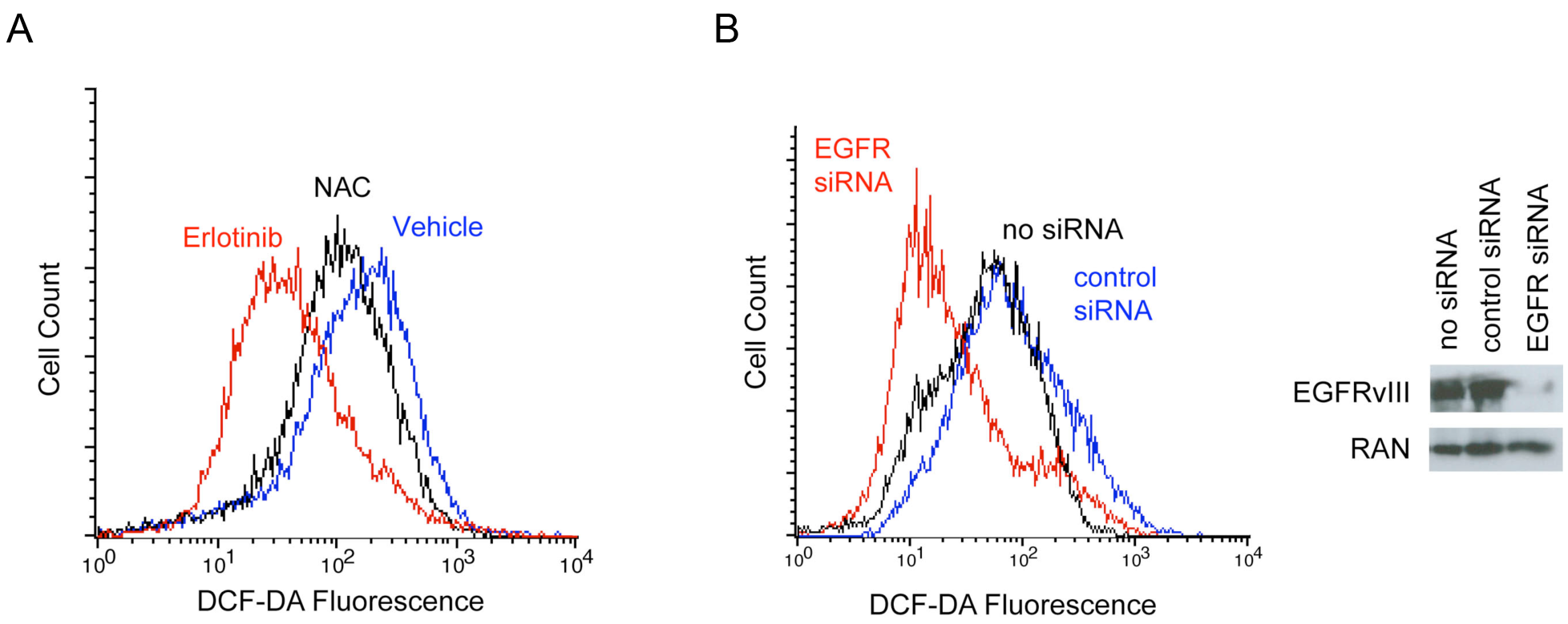

Supplement: Figure S3 — EGFR inhibition or silencing decreased ROS level in U87MG-EGFRvIII cells. (A) EGFR inhibition by erlotnib in U87MG-EGFRvIII cells decreased ROS levels. U87MG-EGFRvIII cells were treated with DMSO or erlotinib (10 mM) or N-acetylcysteine (NAC, Sigma) as a positive control for 48 hours. Cells were harvested, washed with PBS, and incubated with DCF-DA (5 mM) for 15 min. Cells were then washed with PBS and analyzed by FACS. (B) EGFR silencing reduces ROS levels in U87MG-EGFRvIII cells. U87MG-EGFRvIII cells were transfected with siRNA against EGFR or a control siRNA. Cells were cultured for another 72 hours. DCF-DA fluorescence was measured by FACS (left panel). Efficiency of EGFRvIII silencing is shown in the right panel. (0.48 MB TIF) [file pone.0010767.s004.tif]

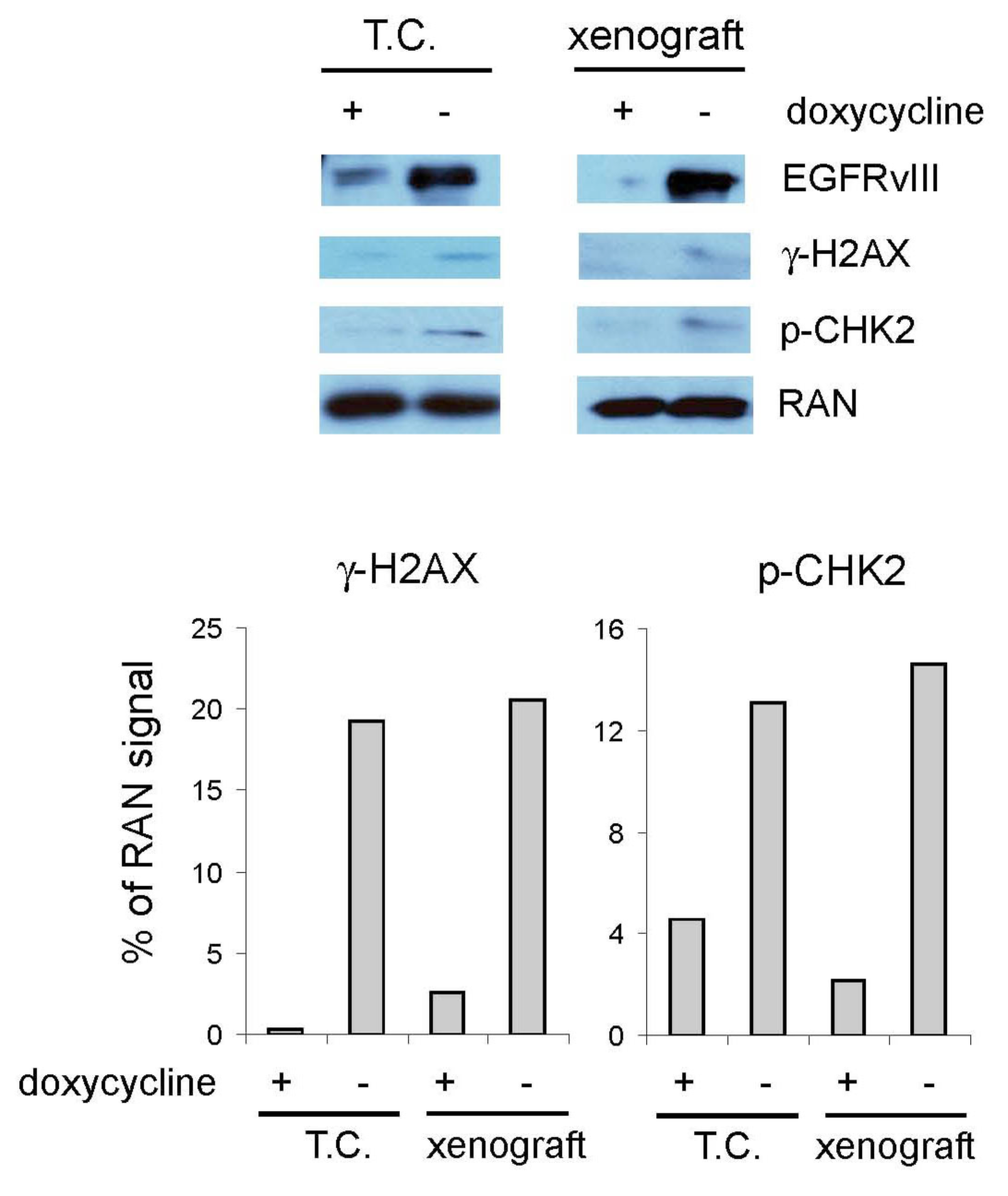

Supplement: Figure S4 — EGFRvIII expression is associated with DNA damage accumulation in U373MG cells. U373MG cells harboring a tet-repressible EGFRvIII construct [1] were treated with doxycycline (1 mM) or vehicle for 72 hours. Cell lysates were prepared and levels of γ-H2AX and p-Chk2 were analyzed (top panel). Intensity of γ-H2AX and p-Chk2 bands were quantified and normalized to the intensity of the RAN loading control (bar graph in the bottom panel). (0.38 MB TIF) [file pone.0010767.s005.tif]

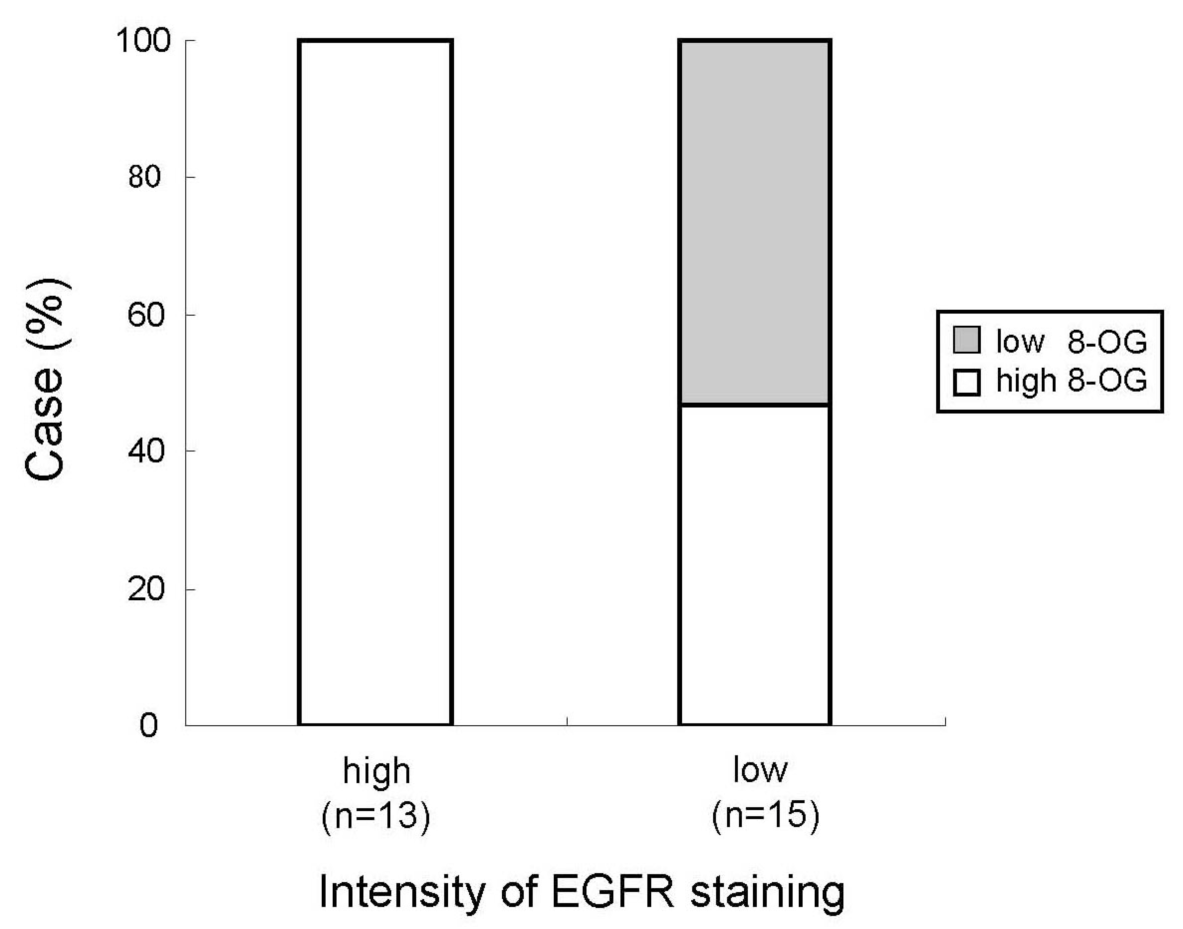

Supplement: Figure S5 — EGFR expression correlates with 8-hydroxyguanosine (8-OG) staining in low-grade gliomas. A low-grade gliomas microarray (US Biomax) was stained for EGFR and 8-OG (see Supplemental Materials and Methods). Samples were stratified into high or low EGFR staining groups. Within each group, the percent of cases with high 8-OG staining is shown in white; low 8-OG staining cases shown in gray. High EGFR staining glioblastomas tend to exhibit high 8-OG staining (p<0.05 by Student's t-test). (0.18 MB TIF) [file pone.0010767.s006.tif]

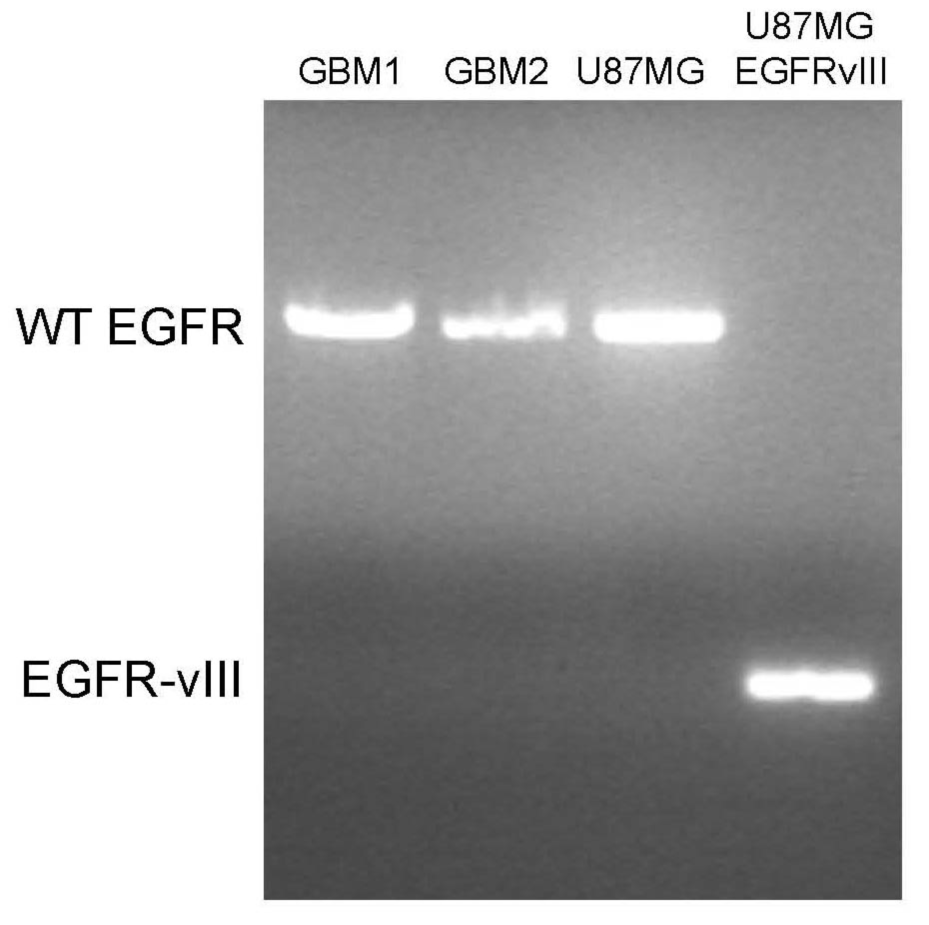

Supplement: Figure S6 — Primary glioblastoma lines used did not harbor EGFRvIII transcript. EGFR transcripts in primary glioblastoma lines were examined by RT-PCR. Ethidium bromide stained gel showing EGFR PCR products from two primary glioblastoma lines, GBM1 and GBM2. mRNAs isolated from U87MG (expressing EGFR) and U87MG-EGFRvIII were used as controls. (0.31 MB TIF) [file pone.0010767.s007.tif]
